# Gait speed and body mass index: Results from the AMI study

**DOI:** 10.1371/journal.pone.0229979

**Published:** 2020-03-10

**Authors:** Maturin Tabue-Teguo, Karine Perès, Nadine Simo, Mélanie Le Goff, Mario Ulises Perez Zepeda, Catherine Féart, Jean-François Dartigues, Hélène Amieva, Matteo Cesari

**Affiliations:** 1 CHU de Guadeloupe, Guadeloupe, France; 2 INSERM 1219, Bordeaux Population Health Research Center, Université de Bordeaux, Bordeaux, France; 3 Equipe LAMIA, Université des Antilles, Pointe-à-Pitre, France; 4 Departamento de Investigacion de Epidemiologia y geriatria, Instituto Nacional de Geriatria, Mexico City, Mexico; 5 Instituto de Envejecimiento, Pontificia Universidad Javeriana, Bogotá, Colombia; 6 Geriatric Unit, Fondazione IRCCS Ca’ Granda, Ospedale Maggiore Policlinico, Milano, Italy; 7 Department of Clinical Sciences and Community Health, University of Milan, Milano, Italy; International University of Health and Welfare, School of Medicine, JAPAN

## Abstract

**Background:**

While physical frailty and malnutrition/obesity (parameters easily measured by a nurse) are not the same, older persons who are malnourished/obese are more likely to be frail and there is a potential overlap between these conditions. The objective was to examine the relationship between gait speed (GS) and body mass index (BMI) in men and women aged 75 years and older.

**Design:**

Cross-sectional analysis.

**Setting, participants:**

Data from the Aging Multidisciplinary Investigation (AMI), a French prospective cohort study with participants randomly selected from the farmer Health Insurance rolls.

**Measurements:**

Usual GS was measured over a 4 meters-track. BMI was categorized using clinical cut-points for European populations: (e.g, <20.0 kg/m^2^; 20.0–24.9 kg/m^2^; 25.0–29.9 kg/m^2^; 30.0–34.9 kg/m^2^; ≥35.0 kg/m^2^).

**Results:**

The current analyses were performed in 449 participants. Mean age was 81 years. Being malnourished/obese was significantly associated with slow GS. Unadjusted and age-adjusted models showed that underweight, overweight and obesity statuses were significantly associated with slow GS for both women (0.83m/s [0.61; 1.04], 0.87m/s [0.72; 1.02], 0.70 m/s [0.41; 0.98], respectively) and men (0.83m/s [0.61; 1.04], 1.11m/s [1.03; 1.20], 0.97m/s [0.75; 1.19], respectively).

**Conclusion:**

Malnourished/obese are associated with slow GS in older persons. These variables could be contributed at comprehensively and complementarily assessing the older person.

## Introduction

Slowness is one of the fundamental characteristics of aging[1] and one of the main components of the decline in physical function. Loss of physical function represents a major public health issue, especially considering the growing number of older persons in Western countries[2,3]. Gait speed (GS) is among the most commonly used instruments for measuring physical performance in population studies of aging[4]. GS is increasingly indicated as an important parameter to measure in the clinical and research evaluation of older persons[2]. It is associated with clinical comorbidity[4] (such as multiple brain lacunae) and subclinical (eg, inflammation[5], oxidative damage[6]) conditions, and predictive of major health-related outcomes (eg, disability, hospitalization, mortality)[7].

The 3C-Dijon and 3C-Bordeaux studies[8] have already shown that GS is specifically associated with the risk of incident dementia[1]. GS is also included in the most common operational definitions of frailty as one of the key criteria to measure[9]. Obesity represents a major priority for healthcare systems as well, due its social, clinical, and economic burdens[10]. In fact, it has shown to significantly predict negative outcome (including disability and mortality). Obesity is an incresingly prevalent disease at all ages, including among older persons[11–14]. Both GS and BMI could be considered as general markers of wellbeing. Although previous studies have demonstrated a U-shaped relationship between body mass index (BMI) and frailty phenotypes[15,16], few others studies have explored the relationship between BMI and GS[17–19] but no study has yet explored this relationship in 75 years and older in rural area. This latter may represent a clinical friendly parameter to adopt in the identification of the "biologically aged" (or frail) individuals.

In the present study, we hypothesize that GS and BMI are associated following a U-shaped relationship (ie, individuals with slow GS are malnourished or obese). While physical frailty and malnutrition/obese (consider as easy-to-access parameter collected by a nurse) are not the same, older person who are malnourished/obese are more likely to be frail and there is a potential overlap between these conditions. The objective of this study was to test whether BMI and 4-meters GS are associated or not among community-dwelling older adults aged 75 years and older. To achieve these goals, we used the Aging Multidisciplinary Investigation (AMI) cohort study data, a French longitudinal population-based cohort.

## Methods

The data used in this study were collected as part of the Agrica-MSA-IFR de Santé Publique, Aging Multidisciplinary Investigation (AMI) study, a French prospective cohort study on health and aging including older farmers living in rural areas. The AMI cohort started in 2007 and included 1,002 older farmers retired from agriculture, living in rural areas. Participants aged 65 years and older were randomly recruited from the Farmer Health Insurance System (Mutualité Sociale Agricole [MSA]). At baseline, trained nurses and psychologists collected information including socio-demographics, self-reported chronic diseases, depressive symptoms, and functional status during face-to-face interviews at the participants’ home. In addition, participants underwent a comprehensive cognitive evaluation. A detailed description of the methodology used for the AMI study can be found in a previous publication[20].

The Ethical Committee of the University Hospital of Bordeaux (Bordeaux, France) approved the AMI study according to the principles embodied on the Declaration of Helsinki and all participants provided written informed consent.

### Body mass index

Standardized measures of weight and size were recorded and used to calculate BMI. BMI was operationalized as the ratio between [weight in kg/height (in m)^2^]. Participants were considered as malnourish and obese if presenting a BMI <21 kg/m^2^ and BMI >30 kg/m^2^, respectively. Indeed, BMI< 21kg/m^2^ is mainly used for the identification of people at risk of malnutrition in the Mini Nutritional Assessment (a validated tool to assess malnutrition and risk for malnutrition among elderly participants of epidemiological studies)[21,22].

### Gait speed

Participants were then asked to walk a distance of 4 meter at their usual pace starting from a still position. GS was operationalized as the ratio between distance and time, and expressed in meters by seconds (m/s).

### Other variables

Disability for the basic activities of the daily living (ADL) and instrumental ADL (IADL) was assessed using the Katz index[23] and Lawton & Brody scales[24], respectively. Cognitive status was measured using the Mini Mental State Examination (MMSE)[25] 17 and depressive symptom by the CES-D[26] 18.

#### Exclusion criterion

We are interested only about a sample of 75 years and older because as people aged 65 to 74 years have no alteration of gait speed in a complementary analyze.

### Statistical analysis

The sample characteristics are presented as the means and standard deviations (+/−*SD*) for continuous variables and as frequencies and percentages for categorical variables. Given the well-established gender differences for body composition and physical performance, all the present analyses were separately conducted for men and women. BMI was categorized using the well-established clinical cut-points for European populations: <20.0 kg/m^2^; 20.0–24.9 kg/m^2^; 25.0–29.9 kg/m^2^; 30.0–34.9 kg/m^2^; ≥35.0 kg/m^2^. Unadjusted and adjusted analyses of covariance were performed to estimate the gender-specific means (and 95% confidence intervals) of GS (in m/s) according to BMI categories. After having provided results from the unadjusted analyses, age-adjusted models were performed. Pearson correlation coefficients were used to examine the associations between BMI and GS. *P*-values < 0.05 were considered statistically significant and 95% confidence intervals (95% CI) are provided. All analyses were performed using SAS, version 9.4 (SAS Institute, Inc., Cary, NC).

## Results

Among the 1,002 participants enrolled in the AMI study, 538 were aged 75 years or older at baseline. We are interested only about a sample of 75 years and older because as people aged 65 to 74 years have no alteration of gait speed in a complementary analyze. The current analyses were performed in 449 (83.5%) participants, after exclusion of 89 (16.5%) individuals with missing data for the main variables of interest (GS or/and BMI).

Table 1 shows that, compared to participants considered in the present analyses, the excluded 89 were older (83.1±5.4 versus 81.0±4.4), had a lower MMSE (20.5±7.9 versus 24.4±4.0) and higher disability in ADLs and IADLs. There were no significant differences for BMI and gait speed between the two groups.

**Table 1:** Main characteristics of the study sample according to participation or missing values.

	Participants N = 449	Non participants N = 89	P*
Age (years)	81.0 (4.4)	83.1 (5.4)	0.0008
Sex (men)	290 (64.6)	51 (57.3)	0.19
Height (m)	1.65 (0.08)	N = 32 1.64 (0.09)	0.54
Weight (kg)	74.6 (14.5)	N = 34 71.4 (14.7)	0.21
Body Mass Index (kg/m^2^)	27.4 (4.3)	N = 30 26.8 (5.0)	0.45
Mini Mental State Examination	N = 440 24.4 (4.0)	N = 88 20.5 (7.9)	<0.0001
CES-D	N = 396 5.2 (7.2)	N = 57 5.9 (7.8)	0.48
Instrumental Activities of Daily Living	N = 444 153 (34.5)	54 (60.7)	<0.0001
Activities of Daily Living	N = 445 33 (7.4)	N = 88 32 (36.4)	<0.0001

Data are expressed as means (standard deviations), or n (percentages).

CES-D: Center for Epidemiologic Studies-Depression Scale

*T-test or chi-square or Fisher test as appropriate

Table 2 shows the socio-demographic characteristics and health status according to gender at the baseline. The mean age of the 449 participants [159 women (35.4%) and 290 men (64.6%)] was 81.2 years (SD 5.0) for women and 80.8 years (SD 4.0) for men. Women had more depressive symptoms (p<.001), lower BMI (p = .02), more disability for IADL (p<.0001) and ADL (p = .006) compared to men. No difference between men and women was reported for age and cognitive status.

**Table 2:** Main characteristics of the study sample according to gender.

	Women 159 (35.4)	Men 290 (64.6)	P*
Age (years)	81.2 (5.0)	80.8 (4.0)	0.46
Height (m)	1.58 (0.06)	1.68 (0.07)	<0.001
Weight (kg)	66.9 (13.5)	78.9 (13.2)	<0.001
Body Mass Index (kg/m^2^)	26.7 (4.9)	27.8 (3.9)	0.02
Mini Mental State Examination	24.4 (3.9)	24.5 (4.1)	0.82
CES-D	6.8 (8.6)	4.3 (6.1)	0.002
Disability on Instrumental Activities of Daily Living	73 (46.5)	80 (27.9)	<0.001
Disability on Activities of Daily Living	19 (12.0)	14 (4.9)	0.006
4-meter gait speed (m/sec)	0.98 (0.42)	1.12 (0.37)	<0.001

Data are expressed as means (standard deviations), or n (percentages).

CES-D: Center for Epidemiologic Studies-Depression Scale

*T-test or chi-square or Fisher test as appropriate

The relationship between the GS and BMI categories in analyses of covariance is presented in Table 3. The unadjusted means of GS were 0.83 (95% CI 0.61–1.04) m/sec in underweight participants, 0.87 (95% CI 0.72–1.02) m/sec in normal BMI, and 0.70 (95% CI 0.41–0.98) m/sec in overweight/obese individuals. After adjustment for age, the reported trends between GS and BMI in categories were largely confirmed. See adjustment for age and comorbidity in complementary analyses (S1 Annexe).

**Table 3:** Results from unadjusted and adjusted analyses of covariance presenting means (and 95% confidence intervals) of 4-meter gait speed according to body mass index (BMI) categories, stratified by gender.

	Unadjusted	Adjusted for age
***Women (n = 159)***		
a. BMI <20.0 kg/m^2^ (n = 14)	0.83 [0.61; 1.04]	0.95 [0.74; 1.16]
b. BMI 20.0–24.9 kg/m^2^ (n = 47)	1.11 [0.99; 1.23]	1.09 [0.98; 1.20]
c. BMI 25.0–29.9 kg/m^2^ (n = 62)	1.00 [0.90; 1.10]	1.00 [0.91; 1.10]
d. BMI 30.0–34.9 kg/m^2^ (n = 28)	0.87 [0.72; 1.02]	0.82 [0.67; 0.97]
e. BMI ≥35.0 kg/m^2^ (n = 8)	0.70 [0.41; 0.98]	0.70 [0.43; 0.98]
P	0.02	<0.001
***Men (n = 290)***		
a. BMI <20.0 kg/m^2^ (n = 7)	0.83 [0.55; 1.10]	0.85 [0.58; 1.12]
b. BMI 20.0–24.9 kg/m^2^ (n = 62)	1.10 [1.01; 1.20]	1.13 [1.04; 1.22]
c. BMI 25.0–29.9 kg/m^2^ (n = 140)	1.16 [1.10; 1.22]	1.16 [1.10; 1.22]
d. BMI 30.0–34.9 kg/m^2^ (n = 70)	1.11 [1.03; 1.20]	1.11 [1.02; 1.19]
e. BMI ≥35.0 kg/m^2^ (n = 11)	0.97 [0.75; 1.19]	0.94 [0.73; 1.16]
P	0.08	<0.001

**Fig 1:**
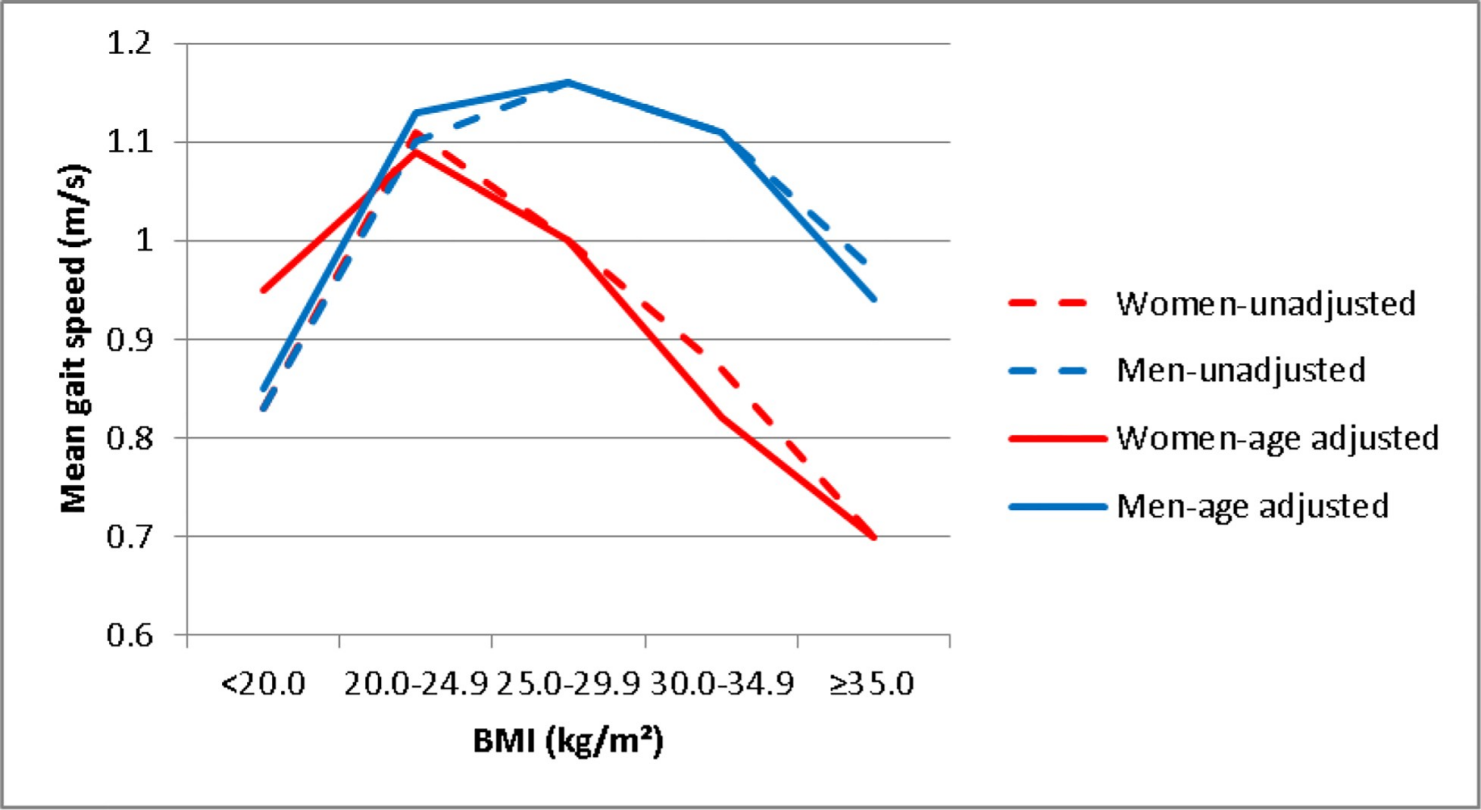
Association between gait speed and BMI. Red lines represent women. Blue lines represent men. Dashed lines show unadjusted results. Continuous lines show age-adjusted results.

GS and BMI (continuous variables) were not significantly correlated (P = 0.16). Non-linear trends between BMI and GS were reported for men and women (Fig 1).

## Discussion

In this cross-sectional analysis of persons aged 75 years and older, we found that GS follows a non-linear pattern across BMI groups. Our findings show that underweight and overweight/obese individuals are significantly slower in GS compared to persons with normal BMI. These relationships are independent of age.

Malnutrition is considered as a core feature of the frailty vicious cycle and contributes to the deterioration of the physical condition (including the mobility capacity) of older people[9].

The elderly population experiences a wide array of physical changes over time. Among these changes, loss of motor neurons, loss of muscle mass and lower aerobic capacity may greatly impact adaptation to everyday life situations. Indeed, they lead to a decrease in muscle strength, which is associated with slower gait speed[27,28] 19^+ (REF)^. Several longitudinal studies suggest a link between BMI and GS[16,29–33]. Our results are partly consistent with these previous reports, but add knowledge in the field under specific aspects. In the present study, we explored the patterns of GS across BMI levels. High and low BMI is here shown to constitute a risk factor for being a slow walker, thus more vulnerability to stressors and exposed to negative outcomes.

Being overweight and obesity have been previously linked to sarcopenia[34]. Thus, individuals with obesity may spontaneously become slow walkers due to the growing balance impairment and physical fatigue. The excess of adipose tissue may alter the optimal ratio with lean mass and affect the quality and function of the skeletal muscle. Consistently, malnourished individuals may reduce their GS because of quantitative and qualitative deficits of the skeletal muscle[5,35].

BMI and GS can be considered two major markers of wellbeing in older people. They indeed provide comprehensive information about the helath status of the individual, even beyond the specific domain they are designed to measure (i.e., nutritional status and mobility function). Our study indicates a tendency [limited size of subjects in the categories (BMI<20 and BMI>30)] to nonlinear association between GS and BMI. Nevertheless, this finding could have an important implication in research. In fact, future works in the field may require the statistical models taking into account the non-linear relationship between BMI and GS.

This study had some limitations. First, the participants were recruited among persons aged 75 years and older living in rural areas. This may introduce a representativeness bias for external validation of our results. Second, the limited sample size may have affected the possibility to conduct more detailed analyses. Third, no conclusion in regard to cause or consequence can be drawn, due to the cross-sectional approach.

A tendency of non-linear relationship between gait speed and BMI is here demonstrated. The two variables are not correlated to one another and may provide complementary information in the comprehensive assessment of older persons.

## Supporting information

S1 Annexe Results from adjusted analyses of covariance presenting means (and 95% confidence intervals) of 4-meter gait speed according to body mass index (BMI) categories, stratified by gender. (DOCX)
