# Supplementary material for: Gait speed and body mass index: Results from the AMI study
Source: PLoS One. 2020 Mar 10;15(3):e0229979. doi: 10.1371/journal.pone.0229979 (PMC7064171; doi:10.1371/journal.pone.0229979)
Supplement: S1 Annexe — (DOCX) [file pone.0229979.s001.docx]

**Annexe 1:** Results from adjusted analyses of covariance presenting means (and 95% confidence intervals) of 4-meter gait speed according to body mass index (BMI) categories, stratified by gender.

|  | Model 1 | Model 2 |
| --- | --- | --- |
| ***Women (n=159)*** |  |  |
| a. BMI <20.0 kg/m^2^ (n=14) | 0.67 [0.32; 1.02] | 0.75 [0.38; 1.11] |
| b. BMI 20.0-24.9 kg/m^2^ (n=47) | 0.88 [0.60; 1.15] | 0.92 [0.64; 1.20] |
| c. BMI 25.0-29.9 kg/m^2^ (n=62) | 0.78 [0.53; 1.03] | 0.83 [0.57; 1.10] |
| d. BMI 30.0-34.9 kg/m^2^ (n=28) | 0.71 [0.43; 1.00] | 0.75 [0.46; 1.04] |
| e. BMI ≥35.0 kg/m^2^ (n=8) | 0.56 [0.17; 0.94] | 0.61 [0.22; 1.01] |
| P | 0.0007 | 0.0007 |
|  |  |  |
| ***Men (n=290)*** |  |  |
| a. BMI <20.0 kg/m^2^ (n=7) | 0.82 [0.51; 1.12] | 0.83 [0.52; 1.13] |
| b. BMI 20.0-24.9 kg/m^2^ (n=62) | 1.06 [0.93; 1.18] | 1.10 [0.97; 1.22] |
| c. BMI 25.0-29.9 kg/m^2^ (n=140) | 1.09 [0.99; 1.19] | 1.10 [1.00; 1.20] |
| d. BMI 30.0-34.9 kg/m^2^ (n=70) | 1.04 [0.92; 1.15] | 1.05 [0.94; 1.16] |
| e. BMI ≥35.0 kg/m^2^ (n=11) | 1.02 [0.79; 1.24] | 1.02 [0.80; 1.24] |
| P | <0.0001 | <0.0001 |

**Model 1**: Adjusted for Mini Mental State Examination, CES-D, diabetes, coronary artery disease,

chronic obstructive pulmonary disease, hypertension, peripheral artery disease, osteoarthritis.

**Model 2**: Adjusted for age + model1

🗹

| **Elements of Financial/Personal Conflicts** | ***TTM/NS** | | **MUPZ** | | **MLG** | | **KP** | |
| --- | --- | --- | --- | --- | --- | --- | --- | --- |
|  | **Yes** | **No** | **Yes** | **No** | **Yes** | **No** | **Yes** | **No** |
| **Employment or Affiliation** |  | **🗹** |  | **🗹** |  | **🗹** |  | **🗹** |
|  |  |  |  |  |  |  |  |  |
| **Grants/Funds** |  | **🗹** |  | **🗹** |  | **🗹** |  | **🗹** |
|  |  |  |  |  |  |  |  |  |
| **Honoraria** |  | **🗹** |  | **🗹** |  | **🗹** |  | **🗹** |
|  |  |  |  |  |  |  |  |  |
| **Speaker Forum** |  | **🗹** |  | **🗹** |  | **🗹** |  | **🗹** |
|  |  |  |  |  |  |  |  |  |
| **Consultant** |  | **🗹** |  | **🗹** |  | **🗹** |  | **🗹** |
|  |  |  |  |  |  |  |  |  |
| **Stocks** |  | **🗹** |  | **🗹** |  | **🗹** |  | **🗹** |
|  |  |  |  |  |  |  |  |  |
| **Royalties** |  | **🗹** |  | **🗹** |  | **🗹** |  | **🗹** |
|  |  |  |  |  |  |  |  |  |
| **Expert Testimony** |  | **🗹** |  | **🗹** |  | **🗹** |  | **🗹** |
|  |  |  |  |  |  |  |  |  |
| **Board Member** |  | **🗹** |  | **🗹** |  | **🗹** |  | **🗹** |
|  |  |  |  |  |  |  |  |  |
| **Patents** |  | **🗹** |  | **🗹** |  | **🗹** |  | **🗹** |
|  |  |  |  |  |  |  |  |  |
| **Personal Relationship** |  | **🗹** |  | **🗹** |  | **🗹** |  | **🗹** |

| **Elements of Financial/Personal Conflicts** | **CF** | | **JFD** | | **HA** | | **MC** | |
| --- | --- | --- | --- | --- | --- | --- | --- | --- |
|  | **Yes** | **No** | **Yes** | **No** | **Yes** | **No** | **Yes** | **No** |
| **Employment or Affiliation** |  | **🗹** |  | **🗹** |  | **🗹** |  | **🗹** |
|  |  |  |  |  |  |  |  |  |
| **Grants/Funds** |  | **🗹** |  | **🗹** |  | **🗹** |  | **🗹** |
|  |  |  |  |  |  |  |  |  |
| **Honoraria** |  | **🗹** |  | **🗹** |  | **🗹** |  | **🗹** |
|  |  |  |  |  |  |  |  |  |
| **Speaker Forum** |  | **🗹** |  | **🗹** |  | **🗹** |  | **🗹** |
|  |  |  |  |  |  |  |  |  |
| **Consultant** |  | **🗹** |  | **🗹** |  | **🗹** |  | **🗹** |
|  |  |  |  |  |  |  |  |  |
| **Stocks** |  | **🗹** |  | **🗹** |  | **🗹** |  | **🗹** |
|  |  |  |  |  |  |  |  |  |
| **Royalties** |  | **🗹** |  | **🗹** |  | **🗹** |  | **🗹** |
|  |  |  |  |  |  |  |  |  |
| **Expert Testimony** |  | **🗹** |  | **🗹** |  | **🗹** |  | **🗹** |
|  |  |  |  |  |  |  |  |  |
| **Board Member** |  | **🗹** |  | **🗹** |  | **🗹** |  | **🗹** |
|  |  |  |  |  |  |  |  |  |
| **Patents** |  | **🗹** |  | **🗹** |  | **🗹** |  | **🗹** |
|  |  |  |  |  |  |  |  |  |
| **Personal Relationship** |  | **🗹** |  | **🗹** |  | **🗹** |  | **🗹** |

^©^MTT: Maturin Tabue-Teguo,NS: Nadine Simo, KP: Karine Perès, MLG: Mélanie Le Goff, MUPZ: Mario Ulises Perez Zepeda,CF: Catherine Féart, JFD: Jean-François Dartigues, HA: Hélène Amieva, MC: Matteo Cesari
